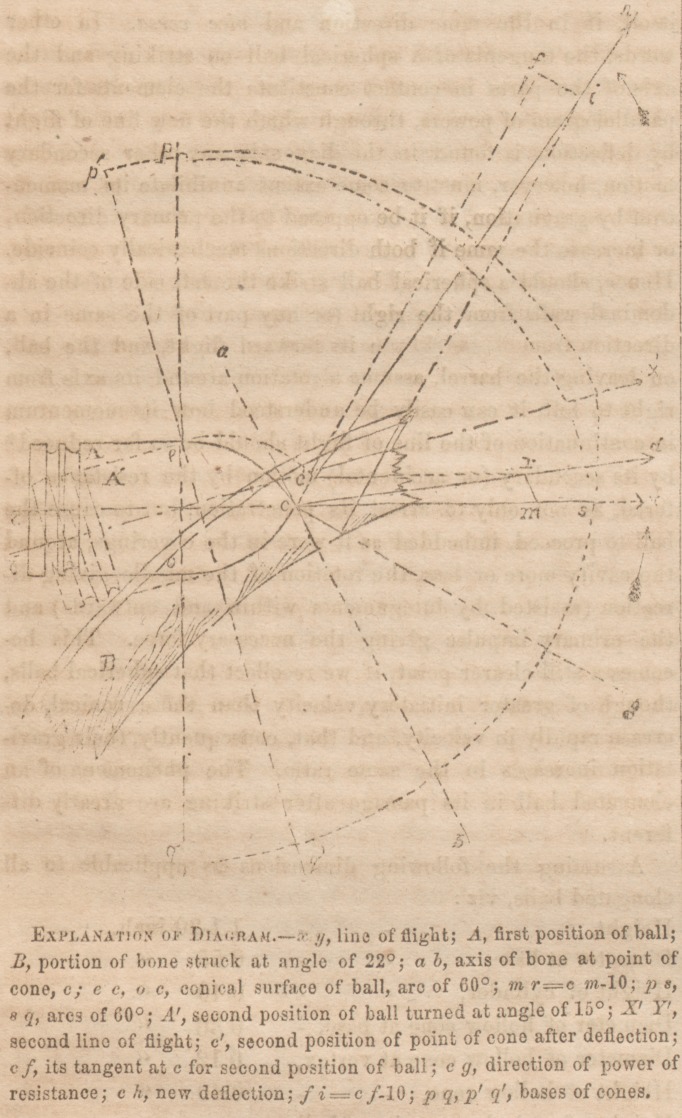# Two Cases of Gun-Shot Wounds, Fracture and Depression of Skull, Resulting in Epilepsy; Trepanning; and Some Remarks on the Nature and Flight of Balls, Spherical and Conical

**Published:** 1864-10

**Authors:** B. Rœmer

**Affiliations:** Surgeon P. A. C. S.


ART. II.—*Two Cases of Gun-Shot Wounds, Fracture and Depression of Skull, Resulting in Epilepsy; Trepanning; and some Remarks on the Nature and Flight of Balls, Spherical and Conical.* By B. RŒMER, Surgeon P. A. C. S.

1.—H. Vandueson, private Company "C," 4th Regiment Texas Volunteers, was admitted into hospital on March 28, 1864, and appears upon the hospital register diagnosticated "Epilepsia consequent upon V. S." His history is as follows: Was wounded on July 3, 1863, the ball traversing and ploughing up that portion of the right parietal bone lying in the angle formed by the coronal and sagittal sutures, anterio-superior aspect; cicatrix formed regularly, and he returned to his command, although he had periodic epileptic fits since September, 1863, which, becoming gradually more intense and of greater frequency, caused his return to hospital in March, 1864, since which time he has been under my charge. The cicatrix was 4½ inches in length, pointing obliquely backwards under an angle of 60° with the coronal suture, deepening and widening in its centre, and presenting a depression of

⅜ inch in depth; the scalp here is radiated, as if the covering had assumed the abnormal condition of the bone beneath. Since his admission the epileptic paroxysms became violent and of greater frequency, from three to four times every month, each composed of a number of successive convulsions. His applications for furlough could not be entertained, because of the possible relief offered by an operation (and in accordance with orders from the Surgeon General S. P. Moore) and on July 18th he was subjected to the trepan. His general condition previous to an attack was marked by dejection of spirits, vertigo and apathy; his bowels habitually costive; appetite wanting; urine scanty; pulse feeble and 60 to the minute; and face pale. Extravasations were supposed to exist, and the trepan was applied with a view to control, if necessary, by a second removal, the whole depressed bone. The bowels having been opened, chloroform was administered, and two incisions were made in form of a T, laying one nearly parallel with the coronal suture upon the upper margin of the parietal bone, about ½ inch from the cicatrix, and uniting with it (at ⅓ its length) the second incision, which extended over the upper third of the cicatrix and behind it. The first measured 3½ and the second 4 inches. On dissecting the two flaps back, which over the cicatrix adhered firmly to the skull, the bone verified its radiated depression. The place of election for trepanning lay at the upper part of the depression, so as to allow the circumference of the trepan to enclose a portion of the fracture. The outer table being very thin, it was easily passed through, the thickness of the inner table, however, demanded a careful progress of the trepan. The bone came away with the trepan, and no adhesions of the dura mater existed. Immediately below and almost in the centre of the opening lay a violet-colored, circular and somewhat convex extravasation, covered by the dura mater, which was divided by a simple cut. (Following the advice of J. Hunter, Lectures, Palmer's edition, vol. 1.) No hemorrhage existed from beneath the skull, and three small arteries of the scalp had been readily controlled without ligation. Careful examination of the inner table beneath the depression (which the convexity of the dura mater rendered difficult) revealed no irregularities. The extravasation being removed, the wound was closed with adhesive straps, cold water dressing applied and a cross bandage. The patient recovered well from the effects of chloroform, and walked about in his quarters on the second day. The wound healed by first intention. He expressed himself two weeks after the operation as free from any unpleasant feelings, such as he experienced since the wound had been received. The condition of his bowels became healthy, his general aspect cheerful, and I have every reason to anticipate a removal of the cause for which the operation was undertaken. The results of this operation, in declining a further trepanning of adjacent parts after removing a portion of skull around, and partly including the fracture of the outer table, rest upon the disclosure of the extravasation, and the cranial opening into which, like as into a safety-valve, the brain and cavelopes could protrude, to the annihilation of any pressure upon itself. The loss of a portion of the outer plane and diplœ *with* depression of surrounding parts and *without* fracture of the internal table, while it is not of unusual occurrence, is yet without anatomical analogy. The position of the wound, or, as I shall aim to show hereafter, the angle under which the line of flight intersected the axis of the parts struck, and the great velocity of the ball; the abnormal thinness of the outer table and the reverse of the inner plane, can alone explain such instances.

2.—The second case, a synopsis of which in relation to its previous history has been kindly furnished me by Dr. R. R. Ritchie, Acting Assistant Surgeon, presents the following characteristics, in giving which I quote from a letter from Dr. R.:

"E. Herring, private Co. "E," 38th Georgia Regiment Volunteers, was wounded at the battle of Sharpsburg on September 17, 1862, and entered this hospital May 18, 1864. According to his statement, he enjoyed perfect health up to the time of receiving his wound. He was struck by a Minie ball in the middle of the right parietal bone, carrying away a considerable portion of both tables of the skull, and from the great press of surgical work at the time, the wound was rather hurriedly dressed on the field, after which he was sent to hospital, where his wound soon closed without having undergone any further examination for spicula or any depressed portions of bone. Soon after the wound had healed he was attacked with epileptic fits, which have continued at uncertain intervals ever since. Upon examination of the cicatrix, a very marked depression was found, and an elastic-yielding sense of touch beneath it. The epilepsy continuing, and the attacks being more frequent, on consultation, an operation (at least exploratory) was decided upon for his relief." In the presence of Surgeons Pride, Rœmer and others, Dr. Ritchie proceeded to place the patient under chloroform, and operated with the trepan, assisted by the above-named surgeons. The incision was in form of a T, the first lying parallel with the sagittal suture, and the second striking it at about its upper third, over the cicatrix and above the point of fluctuation spoken of. The scalp was found adhering to the skull, and abnormal in structure and thickness. The opening through the inner and outer plane was covered with a firm cartilaginous layer, on removing which, considerable hemorrhage from within took place, which was, however, promptly controlled by the application of a heated needle to the orifice of the vessel (no doubt the middle mening. art). The trepan was first applied above this opening and somewhat towards the sagittal and coronal angle, after the removal of which portion of the skull, it being evident that the depression extended further, and the loss of substance (outer table) around the now open skull near the point of fluctuation, precluding the idea of elevating the depressed bone, the trepan was again applied posteriorly and somewhat beneath the first place, at about one inch distance, and the bone having been removed, the edges of the skull between the first and second opening and between either and the point, where both tables had been destroyed by the missile, were taken away by means of Hey's saw; it left thus a truncated triangular opening of about ½ square inch area. Immediately beneath this last locality was disclosed a decided convexity and fluctuation, combined with the peculiar violet discoloration of the dura mater, and upon heavy inspiration a yellowish, serous liquid was seen jetting from a small puncture, which may have been inadvertently made during dissection. This membrane was next carefully divided, and about four ounces of the same liquid discharged. All pressure being removed and the flow of blood arrested, the wound was closed. Adhesive straps alone were found insufficient to bring the edges to adaptation, as the loss of bone and the evacuation of fluid caused the same to collapse, and it became necessary to make use of interrupted sutures. Cold water dressing constituted the treatment, and the patient recovered well from the effects of chloroform. He did unusually well until the morning of the fifth day, when information was brought before daybreak that he had another convulsion. On inspecting the bandages and dressings, they were found bloody and in considerable disorder, and no doubt was entertained that during sleep he had suddenly turned himself on the wounded side, which, if true, would clearly account for this unpleasant feature in the case. Since this accident (as it is esteemed) the patient has been closely watched night and day, and nothing untoward has since occurred. The wound has nearly healed by first intention, and nothing but an accident can now prevent his complete restoration in a few weeks' time.

In this second case the position of the head to that of the direction of the enemy's fire must have been at least under an angle of 65 or 70 degrees, which accords with the statement of Private Herring.

*Remarks.*—The peculiar course of spherical and elongated balls has been often the subject of speculation, and it is presumed that their many apparent irregularities deny explanations of uniform application Whilst some have laid too much weight upon relative velocities, others again have sought to solve the problem through the shape and mechanical bearing of the missile. The following constitute a few ideas on particular instances, selected to suit my purposes, and especially believed to apply to the cases of gun-shot wounds here presented; if necessary, I shall cite other instances treated by myself, and of which I have made careful notes during the time they were under my charge.

The elongated, Minie and Belgian ball, forming as it does a cone of nearly double its height to its base, admits not only of an action like that of a wedge, but also that of a lever, the former directly equal to the products between its weight and momentum of flight, and the latter equal to a quotient between the velocity of the ball and the resistance of the parts struck relative with its angle of penetration. I will premise that all illustrations shall have reference to wounds of the skull only. Let a ball, fired at a given distance, penetrate the thin coverings, the pointed end of the cone will, by its momentum, bury itself to a certain extent into the osseous substance which offers resistance, and if the line of flight be a continuation of the axis of the parts, must either penetrate or become imbedded. Should, however, the trajectory of the ball fail to conform to this direction, different results must follow in a direct ratio with the greater or less deviation from that line.

While the flight of a spherical ball is a compound of a forward and a central rotatory motion, its arrest of progress, on striking, can be multiplied by different angles of deflection, not only because its centre of gravity and motive power are mathematically identical, but also because its rotation around its own axis (without deviation from the line of flight, except such as may be caused by the general laws of gravitation) makes it liable to a deflection in favor of its rotation around itself, if in the same direction and *vice versa.* In other words, the tangents of a spherical ball on striking and the axis of the parts in contact constitute the elements for the parallelogram of powers, through which the new line of flight by deflection is found in the diagonal; any other secondary motion, however, must to some extent annihilate its momentum by gravitation, if it be opposed to the primary direction, or increase the same if both directions mechanically coincide. Hence, should a spherical ball strike the left side of the abdominal walls from the right (or any part of the same in a direction from the right) in its forward flight, and the ball, on leaving the barrel, assume a rotation around its axis from right to left, it can easily be understood how its momentum in continuation of the line of flight should be so far reduced* by its secondary (or accidental) motion by the resistance offered, as not only to arrest its penetration, but to cause the ball to proceed, imbedded as it were in the coverings, around the cavity more or less, the rotation of the missile giving direction (assisted by integuments within and outwards) and the primary impulse giving the necessary force. This becomes a still clearer point, if we recollect that spherical balls, though of greater initiatory velocity than those conical, decrease rapidly in velocity, and that, consequently, their gravitation increases in the same ratio. The phenomena of an elongated ball in its passage after striking are greatly different.

Assuming the following dimensions as applicable to all elongated balls, viz:

| | | |
|---|---|---|
| Height, - - - | 1.1-20 | inch. |
| Diameter at base, - - | 0.55 | " |
| Height of cylinder, - - | 0.40 | " |
| Diameter of hollow cone at base, - | 0.36 | " |
| Diameter of hollow cone at vertex, | 0.12 | " |
| Height of hollow cone, - - | 0.40 | " |
| Height of conical extremity of ball above cylinder, - - | 0.65 | " |

| | |
|---|---|
| then is the cylinder proper (minus hollow cone), | 0.0920 |
| The cone itself, - - - - | 0.1235 |
| And the whole ball, - - - - | 0.216 |

in point of metal, and the point of gravitation (p), lying in the axis $y\ x$ results $yp : xp :: 0.0920 : 0.1235$; its position, in other words, is in the axis nearer to the point of the cone than to its base. The motion of a conical ball, strictly

* Analogous to the peculiar rotation of the "mechanical paradox;" the Australian vomera also owes its peculiar flight to similar circumstances.

speaking, dips forward, whereby its gravitation would be materially increased did not its rotation in the *plane of flight** counteract the same. An increase of forward motion must also be expected from the vacuum around the ball during its flight, and the consequent collapse and forcible entrance of air into the hollow cone, a fact to which we will have occasion to refer hereafter. Hence, while the initial velocity of a conical ball is less than that of a spherical, yet its absolute speed is much greater.

Let A, an elongated ball, strike the skull B under an angle of 22°, and supposing the conical surfaces *ec* and *oc* to form arcs of 60°; let the velocity of the ball be sufficient to penetrate (c) a given depth of the bone, then *ab*, the axis of the bone at *c*, will resist the further progress of the cone, forming with *xy*, line of flight, the elements for the parallelogram of opposing powers; and if *cm*, or the momentum of the ball, be equal to 10 *mr*, or ten times as great as the resistance of *ab*, a deflection of *yx* will take place in the direction of *cd*. Following its new flight for 15°, A′ becomes the new position of the ball with its axis of flight *x′y′*; the point of the cone *c* lying now at *c′*, turned one-fourth of its conical surface. The tangent *cf* measures the resisting force *cg* at *c*, this being the point of contact with the resisting body governing its direction, while the point *c′* demonstrates in its progress the momentum. Allowing again *cf*—10 *fi*, we find *ch* the line of new deflection, by which the point *c′* becomes still more raised above the original axis *xy*, *p′q′*, the second base of the cone *pq*, again turned upon itself until it approximates a parallelism with *xy*. The line so described by *c*, the point of the cone, is an arc, having its most shallow points at entrance and exit and its deepest secant at the centre; the former depending on the amount of resistance offered, and the latter proportional to the momentum of the missile. The result, consequently is—

1. Either a constant deflection towards d and h, and a final departure from the part struck, or

2. An arrest of its flight within the parts in contact, should the velocity of the ball become expended.

Experience has proved the peculiar marks left by balls upon well-supported resistances. That of a cannon ball upon a firm meadow differs from that made in ploughed ground; and a musket ball striking soft or firm parts of the body *caeteris paribus*, creates a wound in conformity with them. In the case of Herring above spoken of, the Minie ball was found in the place deprived of both tables, its point of cone resting upon the brain, and its base nearly horizontal. It had been turned nearly 90°, after creating a wound three inches long in its normal, longitudinal flight (of which fact the wound itself is sufficient proof). Such an occurrence is only possible under conditions agreeing with an arrest of the flight of the ball within the parts struck, and the following coincidences which, if true, would explain many other abnormal positions of elongated balls. After having traversed the scalp, outer table, diploe, and finally the inner table of the skull, the momentum of the ball being exhausted, the hollow cone of the ball received yet the impetus of collapsing air, while, the point of the cone meeting resistance and forming a fulcrum, continued to impart motion to the cylindrical portion of the ball, until the base of the hollow cone became so far turned from the original line of flight as to render further motion by influx of air impossible. Remembering that the point of gravity, in an elongated ball, lies nearer to the vertex of the cone, and that, consequently, it intersects the line of flight above its axis, it follows that the circular opening to the hollow cone presents to the influx of air really an ellipsis, the short diameter of which is a perpendicular to the line of flight, and which becomes shorter as each additional force upon the hollow cone causes it to turn, until it, the ellipsis (and base of cone) assumes a linear aspect. It will be understood that this last condition can only exist, provided the ball has turned 90°, or in other words, stands perpendicular and with its point of cone downwards.

Such a condition will not only arise if the velocity of the ball has been exhausted, but also while the same is yet comparatively unimpaired. On May 31st, 1862, I received the

* Or its helicoid motion around the line of the trajectory.

following case (General Hospital at Richmond): Jul. Mills, Volunteer Aid to Gen. Anderson, was wounded, the Minie ball ranging from above and behind the left ear towards the temporal bone, passing through attollens auris, ploughing up the temporal portion of the great wing of the sphenoid bone, thereby lacerating the attrahens auris, and escaping near the foram. lacer. orbit. superius. The first entrance was near the squamous portion of the temporal bone. By this passage the occipit. artery was cut. About one-half inch beyond its first exit (foramen l. o. sup.) the ball entered again near the supra-orbit. foramen, passing upwards obliquely, and escaped finally about 1½ inches above the ossa nasi near the sinus of the frontal bone. The temporal artery was divided in this second passage. This wound resembled one of incision, and healed by first intention, as reported to Surgeon General S. P. Moore, September 21, 1863. The anatomy of the parts implies a final departure of the conical ball after its first exit, but a quasi directing vis a tergo determined the missile to another line of flight, under an angle not less than from 5 to 10 degrees, whereby the ball re-entered with a sufficient momentum to traverse the skull several inches. On principles as above spoken of, such an occurrence may be accounted for, which otherwise would remain an undissolved problem.

## Figures and Tables

**Figure f1:**